# “Inside‐Out” Central Venous Access approach with infraclavicular exit for right‐sided CRT‐D Implantation in bilateral brachiocephalic and superior vena cava occlusion

**DOI:** 10.1002/ccr3.3980

**Published:** 2021-04-04

**Authors:** Johanna B. Tonko, Stephen A. Black, Christopher A. Rinaldi

**Affiliations:** ^1^ Department of Cardiology St. Thomas Hospital London UK; ^2^ Faculty of Life Sciences and Medicine King’s College London London UK; ^3^ Department of Vascular Surgery St Thomas’ Hospital London UK

**Keywords:** cardiac resynchronization therapy‐defibrillator, central venous access, superior vena cava syndrome, thoracic central vein occlusion

## Abstract

The use of the “inside‐out” approach with an infraclavicular exit site with a dedicated system in the presence of complex central venous occlusion is feasible and safe for the implantation of complex cardiac devices.

## Graphical abstract

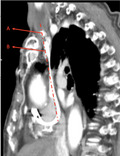

## 1 INTRODUCTION

A significant percentage of patients with implanted cardiac devices need repeated interventions and loss of patency of the thoracic access veins may prevent this. We report a 77‐year‐old man with superior vena cava syndrome undergoing CRT‐D Implantation with an “inside‐out” central venous access approach with an infraclavicular exit.

## 2 CASE PRESENTATION

**FIGURE 1:**
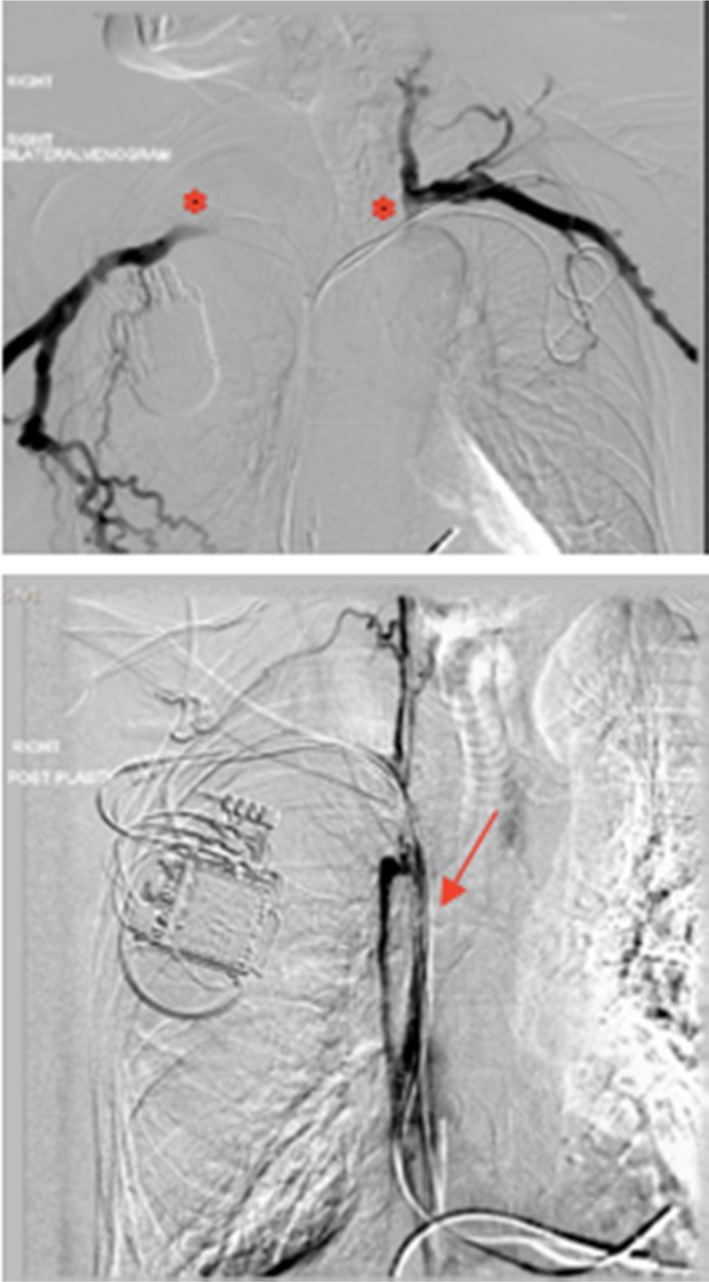
Bilateral DSA of the thoracic central veins 2012 showing bilateral BCV (*) and SVC (arrow) occlusion

In 1999 at the age of 57, the patient received a left‐sided dual‐chamber pacemaker for syncope and 2:1 AV Block. In November 2011, an upgrade to a CRT‐D System was performed for deteriorating LV function (LV EF 30%) with significant interventricular dyssynchrony (paced QRS 166ms) and heart failure symptoms. A pre‐procedure venogram showed a proximal left‐sided subclavian vein stenosis while the right‐sided veins and the SVC were patent and, therefore, a CRT‐D system was implanted on the right‐ and the left‐sided pacing leads were capped and abandoned. Unfortunately, with 5 leads in place and an already occluded left‐sided subclavian vein he developed a superior vena cava syndrome with swelling of his face, bilateral arm edema and cramps especially in the mornings. A venogram confirmed bilateral brachiocephalic and superior vena cava occlusion (Figure 1). To treat his SVC syndrome in February 2012, a laser extraction of his abandoned left‐sided leads was undertaken.

The symptoms transiently improved for a few weeks before they worsened again compatible with recurrent SVC syndrome which was confirmed by a repeated bilateral venogram. A venoplasty with ballooning of the SVC with a 10‐ and 12‐mm balloon was performed in June 2012 (stenting of the SVC was deliberately avoided to prevent jailing the 3 pacing leads in situ). This resulted in a resolution of symptoms. The LV function normalized to 55% in August 2012.

The patient remained well for 7 years but in 2019 suffered from recurrent syncopal episodes due to pacing inhibition as a consequence of noise on his RV shock lead. Investigations at this time also revealed moderate aortic stenosis and a moderate to severe ostial RCA stenosis. In view of noise on the RV shock lead, this was extracted in August 2019 and a new single‐coil shock lead implanted. This was complicated by a pocket infection. A CT venogram showed bilateral brachiocephalic vein and SVC occlusion with reconstitution at the atrial junction corresponding to the most complex type of thoracic central vein occlusion patterns (“Type 4”, classification of the Society of Interventional Radiology)^1^

Extraction of the entire CRT‐D system was performed in October 2019 for pocket infection and a temporary wire was sited due to his 100% pacing dependency.

Two weeks after the extraction, the patient was scheduled for reimplantation of his CRT‐D system on the right side. A preprocedural venogram revealed the right brachiocephalic vein and the superior vena cava were occluded. He went forward to attempted recanalization of the SVC, but this was unsuccessful and, therefore, a leadless pacemaker system (Micra, Medtronic) was implanted via the right femoral vein.

In the following months, the patient reported a general fatigue and restriction associated with a deterioration of his LV function to an LV‐EF of 40%. His cardiac medication at the time included Rivaroxaban 20 mg once daily (for paroxysmal atrial fibrillation), Perindopril 2 mg once daily (uptitration clinically not tolerated) as well as Furosemide 60 mg and Co‐Amilorid 2.5/20 mg once daily to maintain euvolemia, prevent edema in the context of his SVC syndrome and control his recurrent right‐sided pleural effusion. A trial of beta blocker was clinically not tolerated.

The option of a surgical approach with epicardial leads, aortic valve replacement, and CABG was discussed in a multidisciplinary teams (MDT) meeting; however, as the aortic stenosis was only moderate, the patient had no clear symptoms attributable to his moderate‐severe RCA stenosis, and in consideration of the known limitations of epicardial leads in ICD patients, the patient was offered as an alternative a percutaneous CRT‐D reimplantation with the “inside‐out" central venous access approach. Despite not meeting the formal criteria for primary prevention ICD at the time of decision‐making, reinsertion of a primary prevention CRT‐D device (as opposed to downgrading to a CRT‐P) was felt justified in view of the deteriorating LV function, clinical limitations of up‐titration of his heart failure medication and otherwise good overall condition and absence of relevant comorbidities affecting his prognosis.

**FIGURE 2:**
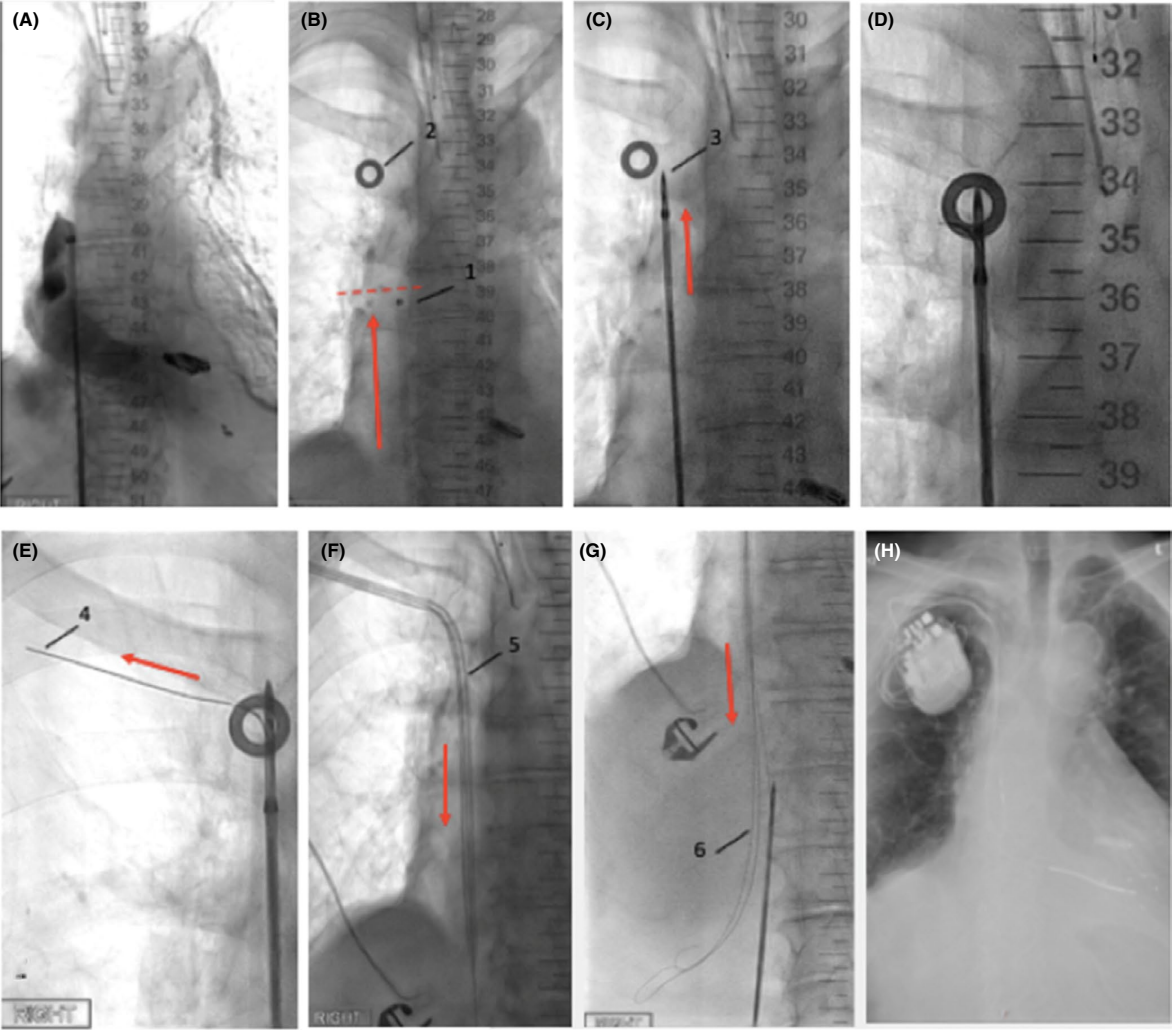
Inside‐Out Venous Access with infraclavicular exit 02/2020: **A,** Venogram demonstrating SVC occlusion, **B,** Working sheath (1) at site of occlusion (horizontal line), radiopaque skin marker (2) at infraclavicular exit target site, **C**, Needle guide (3) and wire advanced through obstructed segment, **D**, fluoroscopy system rotation to adjust tip of device within exit target site, **E,** Opening of tip aligned with target and needle wire (4) advanced anteriorly, **F**, Introducer sheath (5) inserted over externalized needle wire and pulled in and over obstructed segment, **G,** three guide wires (6) inserted over infraclavicular introducer sheath, **H**, henceforth standard CRT‐D Implantation

The procedure (Figure 2) was undertaken under general anesthesia. Ultrasound‐guided right femoral vein access was obtained and a long workstation sheath with a radiopaque marker at the tip was introduced and advanced into the right atrium. A venogram was performed to identify the SVC stump and confirm the site of the occlusion just above the SVC/RA junction.

Subsequently, a Surfacer Device^®^ (Merit Medical) (consisting of a needle guide, needle wire, and a handle) was introduced over the work‐sheath to the occluded segment. A radiopaque marker was placed on the skin in the infraclavicular region to indicate the target exit area for the needle wire. The needle guide was then advanced through the obstruction segment of the SVC and the right brachiocephalic vein under fluoroscopic guidance. The fluoroscopy system was then rotated and adjusted in a RAO/cranial projection until the tip of the device was visible in the exit target. The device tip was rotated so the opening of the tip aligned with the externally placed exit target circle.

The needle wire was then advanced anteriorly through the soft tissue with the indicator on the handle matching the degree of the cranial angulation of the fluoroscopy system. Puncture through the skin with the needle wire was performed at the center of the exit target with externalization and fixation of the needle wire. An introducer sheath was inserted over the externalized wire and pulled through the skin and soft tissue into the vein until the tip passed the occlusion. Following this, the Surface^®^ Device was withdrawn and three Terumo wires inserted through the sheath.

Implantation of a CRT‐D device was then performed in standard fashion using a single‐coil RV lead and positioning the LV lead in a posterolateral vein. Overall, procedure time was 102 minutes and screening time 24.7 minutes (DAP 1774cGycm2). In view of excellent lead measurements after insertion and fluoroscopically well‐sited high voltage lead in a standard RV apical position as well as the increased risk for refractory arrhythmias or thromboembolic events (nonrevascularized coronary artery disease, aortic stenosis, impaired LV function, paroxysmal atrial fibrillation with withhold anticoagulation for 48 hours prior to procedure), a DFT test was not performed. The routine next‐day chest X‐Ray showed unchanged lead positions and no evidence of pneumothorax and the CRT‐D interrogation confirmed stable lead measurements and 100% biventricular pacing with no intrinsic AV conduction. The Micra device (Medtronic) was turned off. The patient was discharged and remains symptomatically well. A follow‐up six months after the procedure showed a mildly improved LV function of 45%, good device function with 99% biventricular pacing and no ventricular arrhythmias.

**FIGURE 3:**
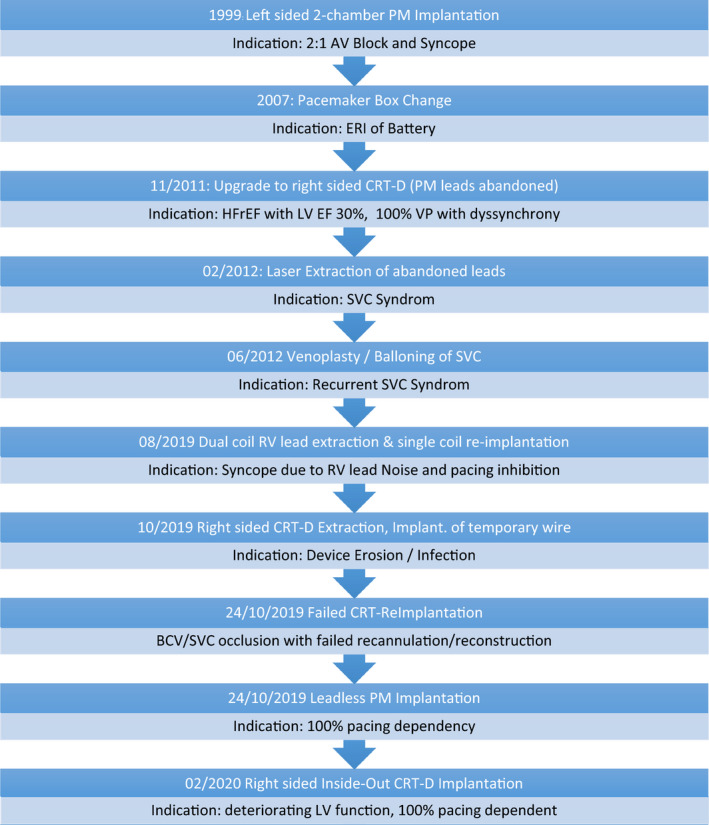
Timeline Device History 1999‐2020

**FIGURE 4:**
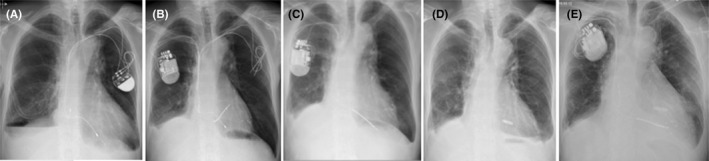
Chest X‐Rays: **A**, 2011 (left‐sided PM), **B**, 02/2012 (right‐sided CRT‐D with dual coil RV shock lead and initially abandoned left‐sided pacing leads, which were later extracted in 06/2012), **C**, 08/2019 (right‐sided CRT‐D with single‐coil RV lead), **D**, 10/2019 (leadless PM), **E,** 02/2020 Re‐implantation of right‐sided CRT‐D, leadless PM in situ. Note chronic pleural effusions

Figure 3 summarizes chronologically the interventions performed over the past 20 years. Figure 4 shows the radiographic documentation of the multiple different cardiac devices implanted along this complex clinical course.

## 3 DISCUSSION

Central venous occlusion as a complication of permanent transvenous pacing has an incidence between 6%‐21%.^2^ In the ICD population, total venous occlusion occurs in 9% and up to 25% of patients display some degree of stenosis at the time of their first generator replacement.^3^ More recently in the population referred for device revision for battery change, device upgrade or extraction excluding those with infection 37.5% were diagnosed with significant central vein stenosis (defined as obvious narrowing with collateral vein development), or occlusion.^4^ In a population referred specifically for lead extraction for infection 32% were found to have access vein occlusion.^5^ Studies indicate that the pathological venous changes occur early after implantation with an incidence of 21% of a venous obstruction > 50%^6^ or occlusion^7^ at 3‐6 months after device implantation. Although venous stenosis and occlusions are commonly identified in fluoroscopy thankfully only 2%‐6% of these patients are symptomatic.^4^ Anticoagulants or antiplatelet medication have been used to prevent this complication with conflicting results and currently, there is no recommendation for the routine use of antiplatelets or anticoagulants for primary prevention of venous stenosis/occlusion.^4^, ^5^, ^6^, ^7^, ^8^ Venous occlusion is best identified by venography via an ipsilateral peripheral catheter.

In 2018, the “*Society of interventional Radiology Reporting Standards for Thoracic Central Vein Obstruction*” proposed a characterization of venous occlusion based on the pattern, localization, and extent of the obstruction with classification of Type 1 to 4.^1^

Different approaches have been used to treat central venous occlusion, including the use of laser or mechanical recanalization tools with or without lead extraction and venoplasty as described in our patient. However, following extraction for infection without immediate reimplantation regaining access to the vein may be difficult. Other approaches include contralateral implantation with a subcutaneous tunnel to the old pocket, femoral/iliac access with leadless pacemaker, or epicardial surgical placement.^9^

**FIGURE 5:**
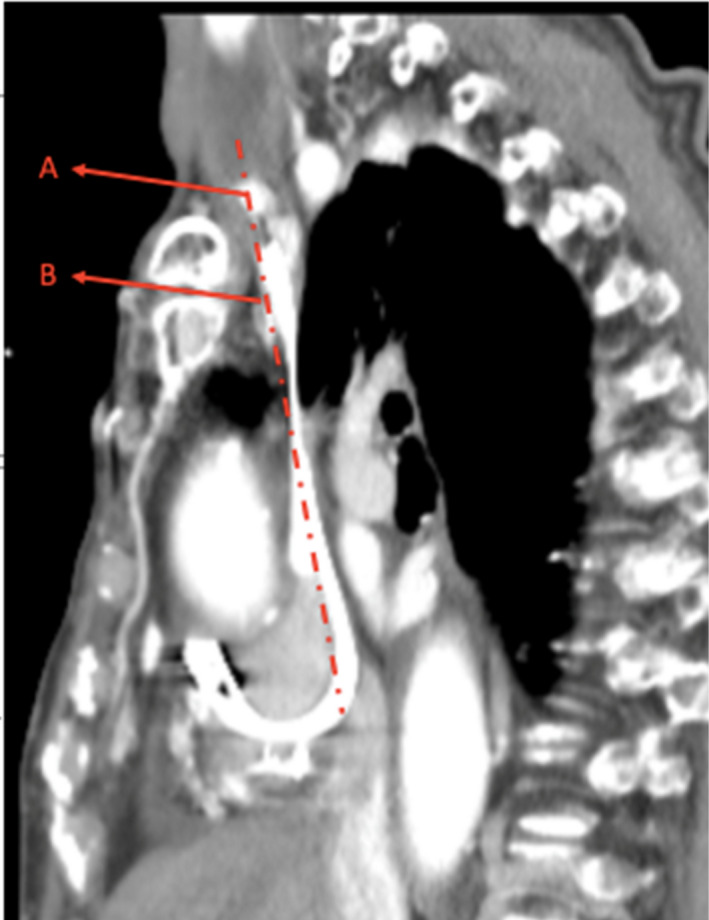
Sagittal plane view of CT Venogram of our patient in 10/2019 pre‐extraction. Vertical line marks VCS/BCV posterior to clavicula. A supraclavicular exit, B infraclavicular exit

The “inside‐out” central venous access for cardiac device implantation was first described in 2011 for patients that could not be recanalized with other existing techniques. This approach accomplishes venous access in a reverse direction from the inside of the vasculature to the outside by tunneling through the occlusion. The first attempts were undertaken with improvised hardware with off‐labeled use of a transseptal dilator, a BRK needle, and a manually sharpened 0.018 inch wire needle for the puncture. A supraclavicular and subclavian exit site were described, where an anteriorly directed needle encounters fat, muscle, and skin, whereas critical structures including lung, hilar vessels and arteries remain posterior to the puncture site. However, the subclavian approach was considered more challenging due to the difficulties of aiming at the narrow rib‐clavicle target window (Figure 5), controlling torque and exit angle, as well as concern about greater proximity to the great arteries compared with the supraclavicular exit.^10^ In 2016 a dedicated percutaneous “inside‐out” access catheter system (Surfacer^®^System, Bluegrass Vascular) designed to provide blunt dissection and better directional control for *supra*clavicular right‐sided access for central veins in patients with obstructed thoracic veins was introduced. In the first‐in‐man study in 2013 (12 patients),^11^ the international post CE surveillance registry^12^ and the first independent multicenter study (39 patients)^13^ the Surfacer^®^System was used for right‐sided supraclavicular access mainly for placement of hemodialysis catheters in the right jugular vein. In the international post CE surveillance registry, only 4% of the interventions were performed for cardiac device implantations. Our case is the first to use the Surfacer^®^System for an infraclavicular exit allowing a standard transvenous lead positioning and prepectoral device implantation preserving the advantages of conventional transvenous lead systems and prepectoral device localization as well as avoiding subcutaneous tunneling of the leads from a supraclavicular position or the risks of thoracotomy based procedures.

The original case series describing the inside‐out procedure in 2011 using improvised and manually modified equipment included 4 successful left‐sided device insertions.^10^ To our knowledge no cases of left‐sided infra‐ or supraclavicular exit using the Surfacer^®^System have been described.

## 4 CONCLUSION

Our case demonstrates the use of the “inside‐out” with a dedicated system with an infraclavicular exit site in the presence of complex central venous occlusion is feasible and safe for the implantation of complex cardiac devices.

## 5 ETHICS STATEMENT

Written informed consent for publication was obtained from the patient.

## CONFLICT OF INTEREST

None declared.

## AUTHOR CONTRIBUTION

JBT: wrote the manuscript. CAR and SAB: critically reviewed and approved the final manuscript version for submission. All authors: participated in the procedure and the management of the patient.

## Data Availability

Case‐related data available on request due to privacy restrictions.
